# Revisiting absent trails: Spatial memory of trail pheromones in termites

**DOI:** 10.1371/journal.pone.0355468

**Published:** 2026-08-12

**Authors:** Yoshihiko Ohzawa, Yukio-Pegio Gunji

**Affiliations:** 1 Global Education Center, Waseda University, Tokyo, Japan; 2 Department of Intermedia Art and Science, Waseda University, Tokyo, Japan; USDA Agricultural Research Service, UNITED STATES OF AMERICA

## Abstract

Termites are social insects that coordinate cooperative behavior through pheromones. Trail pheromones play a central role in foraging and recruitment. Traditionally, termite navigation has often been modeled as a reflexive response to the current gradient of pheromone concentration, implying a stimulus-driven, taxon-type mechanism without necessarily requiring internal spatial representation. However, it remains unclear whether termites can form and utilize spatial information about the location of chemical cues. In this study, we investigated whether previous contact with a trail pheromone can bias termite movement after the pheromone-marked substrate has been removed. In the primary immediate test, entries into the exact target-side circle were not significantly higher in the pheromone condition, indicating that the effect was not a strong immediate return to a precise point. However, across the extended test phase, pheromone-exposed termites showed more entries and inward excursions toward the area where artificial trail pheromone had previously been presented, especially when broader target zones were considered. Together, these results suggest that termites can retain or use coarse spatial information about a previously encountered trail-pheromone cue, resulting in broad spatial bias rather than accurate point revisitation. Our findings extend the traditional view of trail pheromones as purely immediate stimuli by suggesting that pheromone experience can influence later movement after cue removal. This provides a cautious basis for considering how chemical cues may contribute to locale-like navigation in termites.

## 1 Introduction

Termites are highly social insects that exhibit cooperative behaviors such as collective foraging, nest building, and corpse removal. These behaviors are regulated by multiple signals, including chemical, tactile, vibrational, and acoustic cues [1–3]. Pheromone-based signaling, in particular, has been a central focus in models of termite foraging and tunnel construction, as it enables decentralized coordination through trail-mediated interactions.

Among these chemical cues, trail pheromones play a central role in shaping foraging paths and recruiting nestmates [4]. Many behavioral models assume that termites determine their movement direction based on the current gradient of pheromone concentration [5–8]. Such behavior is typically classified as a taxon response—a form of stimulus-driven sensorimotor behavior that does not necessarily involve internal representations of space or past experiences [9–12].

In contrast, other social insects such as ants and bees exhibit locale-like cognition, relying on learned spatial knowledge, landmarks, or route memory to guide their navigation [13,14]. In ants, in particular, interactions between learning and the use of trail pheromones have been demonstrated; for example, ants can modulate their trail-following behavior based on learning [15], and trail pheromones can enhance learning efficiency [16,17].

While spatial cognition in termites has received relatively little attention, accumulating evidence suggests that they may possess more complex navigational and learning abilities than previously assumed. For example, termites can use optical and pheromonal orientation and remember homing distance in open-field conditions [18]. In tunnel construction, termites tend to dig in directions that extend away from the tunnel origin, indicating the use of idiothetic information to maintain a global away vector under conditions designed to minimize external cues [19]. Termites are also capable of identifying shortcuts, suggesting some form of spatial learning or route optimization [20]. Moreover, laboratory experiments have demonstrated that termites can form associations through classical conditioning [21] and display learned avoidance in Y-maze tasks [22], further supporting their capacity for learning.

However, while these studies demonstrate that termites are capable of associative learning, they have not addressed whether such learning extends to the spatial location of trail-pheromone cues. Conversely, research on spatial behavior—particularly in tunnel construction—has focused on innate or rule-based movement patterns and has rarely examined whether termites can retain and utilize the spatial location of external chemical cues after those cues have disappeared.

In this study, we investigate whether termites show a spatial bias toward the location of a previously presented pheromone trace after its removal. Such behavior would differ from immediate trail following and would suggest that prior pheromone experience can influence subsequent movement even in the absence of the pheromone-marked substrate.

Our aim was to determine whether trail pheromone experience can contribute to later spatial behavior. Because the behavioral effect may be broad rather than point-specific, we interpret the results in terms of coarse spatial bias rather than precise memory for a specific pheromone point.

## 2 Materials and methods

### 2.1 Termite collection and maintenance

Termites (*Coptotermes formosanus*) were collected on April 24–25, 2026, from four field colonies on Iriomote Island, Okinawa Prefecture, Japan. Only worker individuals were used; each termite was used only once. The termites were kept in lidded plastic containers at $28\pm {1\;}^{\circ }C$, with regular spraying to maintain humidity, and were provided with wood from their respective colonies but no additional food. The tree species from which the wood originated was not identified.

### 2.2 Experimental setup

As in previous studies observing termite walking patterns, experiments were conducted in a glass Petri dish (radius 42.5 mm) [23,24]. Dishes were reused after washing with detergent and water and wiping with lint-free laboratory wipes. JETSTREAM 5 mm ballpoint pen ink (Mitsubishi Pencil Co., Ltd.) was used as an artificial trail pheromone. Ballpoint pen ink contains components of termite trail pheromones and has been used in behavioral experiments [25–27].

The experimental room was maintained at $28\pm {1\;}^{\circ }C$ under low-light conditions to reduce visual stimulation to the termites. Videos were recorded with a Panasonic HDC-TM700 digital video camera at 29 frames per second and a resolution of 1920×1080 pixels. The camera was fixed vertically above the Petri dish. Eight arenas were recorded simultaneously, and the arenas were arranged so that trials were not aligned to a single room direction.

### 2.3 Experimental conditions

**Fig 1:**
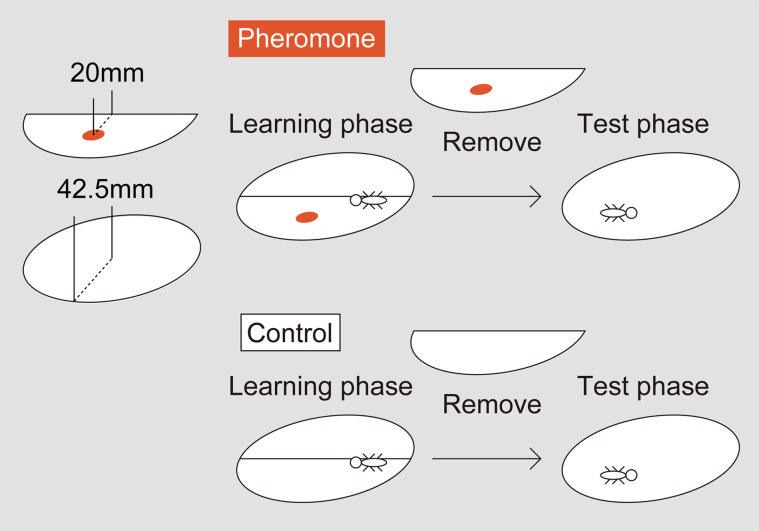
Overview of the experiment. In both the pheromone and control conditions, termites walked during a learning phase with a semicircular sheet of copy paper overlaid on a circular sheet. The semicircular sheet was removed before the test phase. In the pheromone condition, a small dot of artificial trail pheromone was applied to the semicircular sheet. In the control condition, the corresponding semicircular sheet was blank.

Two conditions were used: the pheromone condition and the control condition (Fig 1). A different individual was used for each trial. In both conditions, circular and semicircular sheets of neutral copy paper (radius 42.5 mm) were used to cover the Petri dish surface. The circular sheet was placed on the dish, and the semicircular sheet was layered on top of the circular one.

In the pheromone condition, a small ink dot (radius 1 mm) was marked on the semicircular sheet. To standardize the amount of ink as much as possible, the dot size was fixed by filling a circle of the same radius with the same ballpoint pen in each trial. Experiments were started approximately 10 min after the ink was applied. The dot was positioned 20 mm from the center of the Petri dish, along a line perpendicular to the chord that defines the flat edge of the semicircle. A dot was used rather than a line because a point stimulus defines a single location for testing whether subsequent movement is biased toward the former pheromone location. In the control condition, the same paper arrangement was used, but no ink dot was applied to the semicircular sheet. Thus, the two conditions differed only in whether the semicircular paper contained the artificial pheromone dot.

The side covered by the removable semicircular sheet is hereafter referred to as the target side, and the other half of the arena as the opposite side. In the pheromone condition, the artificial pheromone dot was located on the target side; in the control condition, the target side was defined by the same semicircular-sheet geometry but contained no ink dot. Before exclusions, 48 termites were tested in each condition. The analyzed dataset contained 88 workers: colony 1, 10 control and 10 pheromone; colony 2, 11 control and 10 pheromone; colony 3, 12 control and 11 pheromone; colony 4, 12 control and 12 pheromone. As exceptional procedural issues, individuals were excluded when they entered beneath the semicircular paper during the learning phase or when body parts were damaged during handling before the trial, such as during transfer from the rearing container to the arena.

#### 2.3.1 Pheromone condition.

At the start of each trial, a plastic cylinder with a diameter of 1 cm was placed near the dot position. A termite was introduced into the cylinder, and the cylinder was removed after 5 s. This procedure aligned the initial position across trials and, in the pheromone condition, ensured that the termite encountered the artificial pheromone at the beginning of the trial. Because of the size of the cylinder and the body length of the termite, the termite necessarily overlapped the ink dot in the pheromone condition.

Tracking continued for at least 10 min (the learning phase). While the termite walked on the opposite side, the semicircular paper was carefully removed to avoid disturbance. The learning phase was set to at least 10 min; because removal required the termite to be away from the semicircular sheet, the actual duration ranged from 10 to 12.5 min. Tracking then continued for 30 min (the test phase). The test phase was divided into immediate test (0–10 min after removal), delayed test 1 (10–20 min), and delayed test 2 (20–30 min).

### 2.4 Data acquisition and analysis

Videos were tracked using Trex [28] at 29 frames per second. Due to image noise under low-light conditions, coordinates were manually corrected using AnimalTA [29] based on visual inspection.

The primary behavioral measure was the number of entries into the target-side circle. The target circle was centered at the target point, which was the ink-dot position in the pheromone condition and the corresponding geometric position in the control condition, 20 mm from the arena center, with a radius of 5 mm. The primary test interval was the immediate test phase (0–10 min after paper removal), following the test duration used in the previous experiment. The extended test phase (0–30 min) was also analyzed to examine whether pheromone exposure produced a longer-lasting spatial bias that was not limited to the first 10 min after cue removal.

Entry counts were analyzed using mixed models with condition and total activity as fixed effects and random intercepts for colony and individual. For analyses spanning multiple test bins, phase was also included as a fixed effect, and condition-by-side models were used when target-side and opposite-side entries were analyzed together. Analyses were conducted using Python, and mixed models were fitted with statsmodels [30]. Additional analyses were treated as supplementary and included the center-directed vector analysis, learning-test correlations, occupancy ratios, and sensitivity analyses; these are reported in S1 Text. False discovery rate (FDR) correction was applied to families of supplementary tests, and corrected *q* values are reported in supplementary output tables.

Termites showed strong thigmotaxis and spent much of their time walking near the arena edge. To distinguish departures from this wall-following pattern from ordinary peripheral walking, excursion events were defined as contiguous trajectory segments that started when the termite crossed from *r* > 35 mm to r≤35 mm and ended when it left that zone. This analysis was introduced to detect a more diffuse directional tendency than exact entry into the 5 mm target circle. For each event, we recorded the onset side, the side of the closest point to the arena center, whether the event entered target-side or opposite-side circles, which target was entered first, and the minimum distance to each target. The first-entered target was used to distinguish the initial return-like component of an excursion from subsequent movement associated with the same excursion; for example, if a termite moved from the periphery into the target-side circle, then into the opposite-side circle, and then back to the periphery, only the first target-side entry was counted as the first-hit event. Target proximity bias was defined as the minimum distance to the opposite-side target minus the minimum distance to the target-side target, so positive values indicate that an excursion approached the target-side point more closely than the opposite-side point. In addition to the 5 mm target radius used for the primary entry analysis, we examined broader target radii of 10 and 15 mm to evaluate whether the effect reflected coarse spatial bias rather than precise point revisitation.

## 3 Results

**Fig 2:**
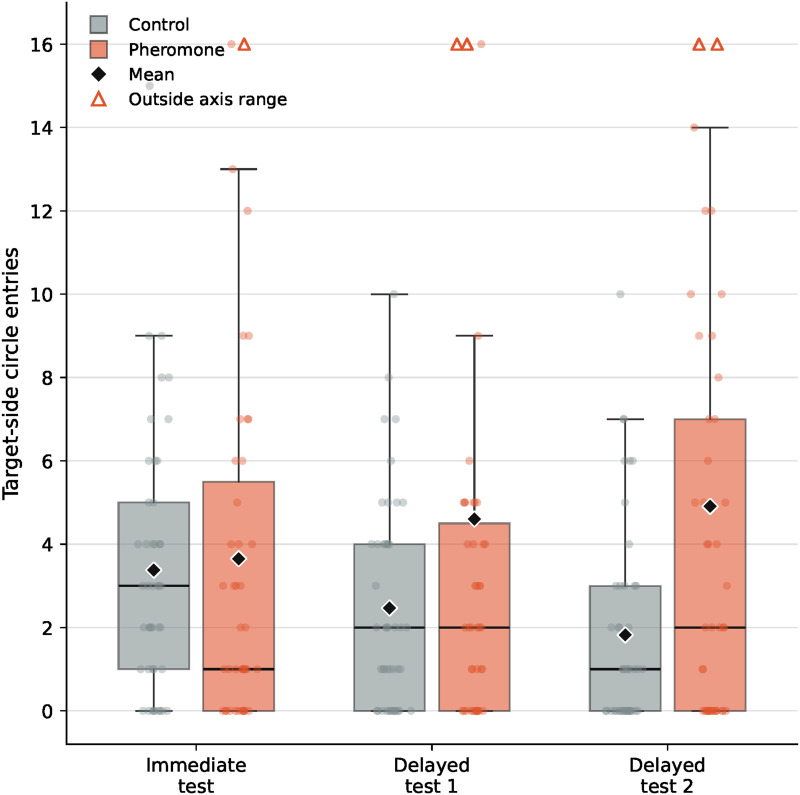
Entries into target-side circles by phase and condition. Boxes show the interquartile range, horizontal lines show medians, whiskers show 1.5 times the interquartile range, circles show individual termites, and black diamonds show means. Upward triangles at the top of the axis indicate individual values above the plotted range. The target side refers to the side covered by the removable semicircular sheet.

Fig 2 presents the primary analysis of entries into target circles in the experiment.

### 3.1 Target entries during the immediate and extended test phases

In the immediate test phase, target-side entries were similar between conditions (control mean = 3.38; pheromone mean = 3.65; Fig 2). A mixed model controlling for total activity and including colony and individual random effects did not detect a significant pheromone effect (β=0.36, SE = 0.82, *z* = 0.44, *p* = 0.663). Thus, the primary immediate test did not provide robust evidence that pheromone-exposed termites revisited the exact former target point more frequently than control termites.

Across the extended 0–30 min test phase, the mean number of target-side entries was higher in the pheromone condition than in the control condition. When only target-side entries were analyzed, the pheromone effect was positive but did not reach the conventional two-sided 0.05 threshold (β=1.88, SE = 1.10, *z* = 1.71, *p* = 0.088). A broader mixed model that used both target-side and opposite-side entries across the test phase detected a significant condition effect (β=1.61, SE = 0.81, *z* = 2.00, *p* = 0.045). The direction of the mean difference was consistent across the three test bins: target-side entries in the pheromone condition were 3.65, 4.61, and 4.91 in immediate test, delayed test 1, and delayed test 2, respectively, compared with 3.38, 2.47, and 1.82 in the control condition. These results indicate that the effect was not a strong immediate return to the exact former target point, but became clearer when behavior was observed over the extended test period.

### 3.2 Excursion events and broader target zones

Because the entry analysis suggested that termites may not precisely revisit the former target point, we examined whether pheromone exposure altered broader departures from the periphery. Across the extended test phase, pheromone-exposed termites showed more excursions into the inner region than control termites (β=19.55, SE = 9.53, *z* = 2.05, *p* = 0.040). Excursions that started on the target side were also more frequent in the pheromone condition (β=11.20, SE = 5.35, *z* = 2.10, *p* = 0.036), as were excursions whose closest point occurred on the target side (β=10.67, SE = 5.41, *z* = 1.97, *p* = 0.048).

**Fig 3:**
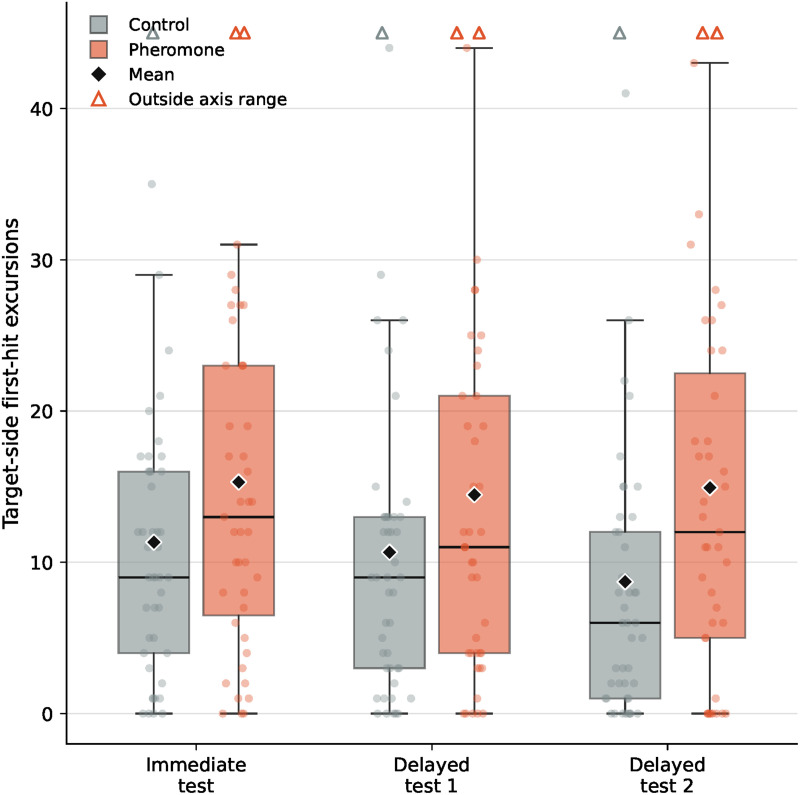
First-hit excursion events into the target-side circle using a broader 15 mm target radius. Boxes show the interquartile range, horizontal lines show medians, whiskers show 1.5 times the interquartile range, circles show individual termites, and black diamonds show means. Upward triangles at the top of the axis indicate individual values above the plotted range. This analysis evaluated whether the pheromone effect was better described as a coarse spatial bias than as precise point revisitation.

**Fig 4:**
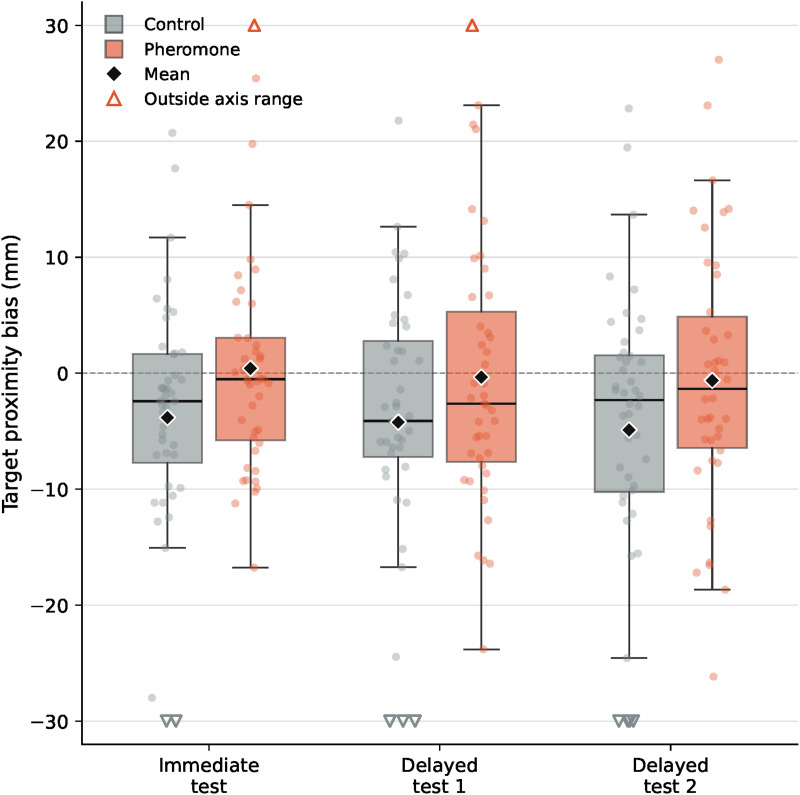
Target proximity bias. Boxes show the interquartile range, horizontal lines show medians, whiskers show 1.5 times the interquartile range, circles show individual termites, and black diamonds show means. Upward triangles at the axis limits indicate individual values outside the plotted range. Positive values indicate that excursions approached the target-side point more closely than the opposite-side point.

For excursion events, entering the exact 5 mm target circle was a stricter criterion than approaching the broader target region. Target-side circle-hit events showed a positive pheromone effect but were not significant in a two-sided test (β=1.70, SE = 1.01, *z* = 1.69, *p* = 0.092). However, target proximity bias was significantly higher after pheromone exposure (β=4.27 mm, SE = 2.10, *z* = 2.03, *p* = 0.042), indicating that excursions approached the target-side circle more closely than the opposite-side circle in the pheromone condition. When the target radius was broadened to 15 mm, first-hit events into the target-side circle were significantly higher in the pheromone condition (*p* = 0.037; Fig 3), and the condition-by-side comparison also supported a stronger target-side bias after pheromone exposure (*p* = 0.039). Target proximity bias showed the same interpretation in a distance-based form: positive values indicate that excursions approached the target-side circle more closely than the opposite-side circle (Fig 4).

### 3.3 Supplementary analyses

The center-directed vector analysis is reported as a supplementary check in S1 Text. It did not provide stronger evidence than the target-entry and excursion analyses.

Learning-test relationships, occupancy ratios, and a sensitivity analysis excluding pheromone-condition termites with no learning-phase target entry are also reported in S1 Text. These analyses were examined as checks on the main analysis and did not provide stronger evidence for precise point memory than the target-entry and excursion analyses.

## 4 Discussion

This study provides evidence that prior exposure to an artificial trail-pheromone point can bias later termite movement after the pheromone-marked substrate has been removed. Whereas previous studies have examined navigation during tunnel construction or associative learning in other contexts, our study focused on navigation after experience with an artificial pheromone trace. The results do not demonstrate precise revisitation of a specific point, but they suggest that termites may retain or use coarse spatial information about the former pheromone-associated region.

As discussed in the Introduction, trail pheromones have traditionally been understood as stimuli that elicit immediate, reflexive responses, a taxon-type mechanism [1,4]. The present results suggest that trail-pheromone experience may also influence subsequent movement after the pheromone substrate has disappeared. This does not mean that termites form precise locale-type representations comparable to those described in ants or bees [12,14]. Rather, the results indicate that chemical experience and spatial exploration may interact even in a simple planar arena.

The coarse nature of the bias admits at least two interpretations. First, termites may retain only approximate spatial information about the region where the trail-pheromone cue was encountered and then rely on direct chemical information only after returning near the cue. Under this interpretation, the observed bias reflects a limit on the spatial precision of pheromone-associated memory. Second, the broad bias may itself be an adaptive search strategy: rather than returning to the exact point, termites may search around the former pheromone location, increasing the probability of reacquiring a displaced or discontinuous trail or locating resources connected to it. These alternatives are not mutually exclusive, because selection for efficient search could favor spatial responses that are intentionally broad rather than point-specific. The present experiment cannot distinguish between limited spatial precision and an adaptive broad-search strategy, and future experiments should manipulate cue uncertainty, cue reappearance, and the ecological value of the cue-associated location.

Trail pheromones are generally considered means for coordinating movement toward resources, nest sites, or routes rather than goals in themselves [1,4]. Nevertheless, if termites bias later search toward the former location of a pheromone cue, the cue may function as a spatially localized predictor of behaviorally relevant routes. This interpretation does not require assuming an explicit metacognitive representation that “pheromone trails lead to food or nests.” A simpler account is that experience with a pheromone cue adaptively increases subsequent search near the cue’s former location. Even under this cautious interpretation, the result goes beyond a binary response in which termites simply follow a currently detected pheromone and ignore it when absent. Instead, trail pheromone may act both as an immediate taxon-like stimulus and as a cue that becomes linked to a coarse spatial location.

Termites show directional, distance-related, and route-related behavior in other contexts, including homing in open-field conditions, tunnel propagation, and route optimization [18–20]. The distinctive feature of the present study is that a localized chemical cue was first presented and then removed during an open-arena trial, allowing us to ask whether movement remained spatially biased after the cue itself was absent.

Nonetheless, several limitations of this study should be noted. First, this study focused exclusively on worker termites. Whether soldiers or reproductives exhibit similar behavior remains unknown. We also did not determine worker sex. Sex-related differences in chemoreception or learning are unlikely to be the primary driver in this worker-based assay, but they cannot be excluded. Moreover, although termites are social insects, this study was limited to the behavior of individual workers. It remains an open question whether similar pheromone-associated spatial biases emerge in group contexts, where interactions among individuals may influence spatial strategies.

Second, we used commercially available ballpoint pen ink as a substitute for natural trail pheromone. Since the ink contains compounds other than the identified active pheromone components, these additional substances might have influenced termite behavior or learning. The ink dot was applied before the semicircular sheet was placed over the circular sheet, and no visible bleed-through was observed from the reverse side of the marked sheet. Although we did not chemically quantify possible transfer of ink components from the removable semicircular sheet to the underlying circular sheet, several aspects of the result argue against a simple direct-response interpretation. The clearest effect appeared as a coarse target-side bias across the extended test phase rather than as a strong immediate increase in exact target hits after removal. If residual ink transfer to the underlying sheet were the dominant driver, a more immediate and spatially precise response would be expected. Nevertheless, chemical transfer cannot be excluded completely without chemical analysis or a purified-pheromone control. Future studies should employ chemically purified pheromones, quantify possible substrate transfer, or analyze the ink’s composition in detail to determine stimulus specificity.

In summary, this study suggests that termites can show a coarse spatial bias toward a previously pheromone-associated region after cue removal. By moving beyond a purely immediate stimulus-response view of trail pheromones, these findings invite further investigation into how chemical cues may contribute to locale-like navigation and spatial exploration in termites.

## Supporting information

S1 Text Supplementary analyses. Center-directed vector analysis, learning-test relationships, occupancy ratios with FDR correction, and sensitivity analysis excluding pheromone-condition termites with no learning-phase target entry.
(PDF)
