# Supplementary material for: Revisiting absent trails: Spatial memory of trail pheromones in termites
Source: PLoS One. 2026 Aug 12;21(8):e0355468. doi: 10.1371/journal.pone.0355468 (PMC13466029; doi:10.1371/journal.pone.0355468)
Supplement: Supplementary file 1 [file pone.0355468.s001.pdf]

S1 Text. Supplementary analyses

Purpose

This file reports supplementary analyses for the experiment. The analyses include the center-directed vector analysis, learning-test relationships, occupancy ratios, and a sensitivity analysis excluding pheromone-condition termites with no learning-phase target entry. These analyses are provided to document checks on the main target-entry and excursion-event analyses and should not be interpreted as primary evidence for precise point memory.

Center-directed vector analysis

Purpose

The center-directed vector analysis was used to detect movements from the arena periphery toward the inner arena. Because the main manuscript now focuses on target-side entries and excursion events, this analysis is retained for transparency and continuity with this analytical approach, but it is not used as the primary evidence for the main conclusion.

Definition

A center-directed vector was defined for each inward movement from the arena periphery. The periphery was defined as the region more than 35 mm from the center of the Petri dish. Each vector started when the termite first reached a distance of 35 mm from the center and

ended at the closest point to the center before the termite left the inner region. The endpoint side was classified as target-side or opposite-side using the same coordinate definition as in the main text.

Statistical analysis and results

The supplementary count analysis tested whether pheromone exposure increased the number of target-side endpoint vectors during the test phase. The model included condition, phase, total vector count, and random intercepts for colony and individual. A second analysis examined the target-side endpoint ratio, again controlling for phase and total vector count.

Center-directed vector counts did not show a significant pheromone effect in the count model (estimate = 1.74, SE = 2.75, z = 0.63, p = 0.526; Table A). The target-side endpoint ratio showed weak evidence in the expected direction, but the two-sided test did not reach the conventional 0.05 threshold (estimate = 0.066, SE = 0.035, z = 1.89, p = 0.059; Table B). Thus, this supplementary analysis did not provide stronger evidence than the target-entry and excursion-event analyses reported in the main text.

Learning-test relationships

We examined whether target-side bias during the learning phase predicted later test-phase bias. In the model predicting test-entry bias, learning-entry bias was associated with test-entry bias (estimate = -0.173, SE = 0.087, z = -1.991, p = 0.046; Table C). This relationship was not straightforwardly interpretable as memory for the pheromone point, because the sign and magnitude may also reflect individual differences in overall exploration and side use. In the model predicting delayed-test bias from learning and immediate-test bias, neither learning-entry bias nor immediate-test bias was significant (Table D).

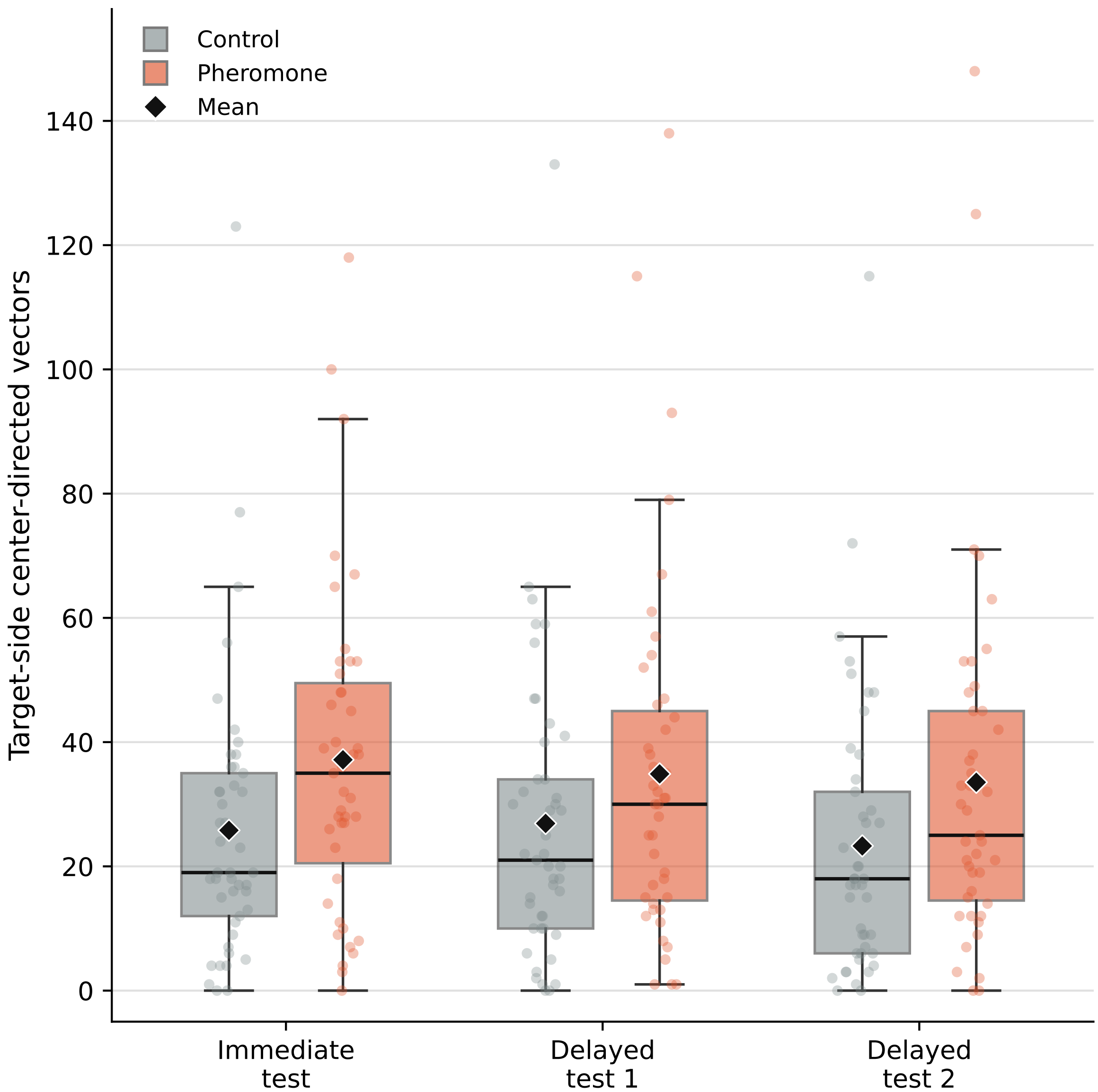

Fig A Center-directed vector counts by condition and phase. Boxes show the interquartile range, horizontal lines show medians, whiskers show 1.5 times the interquartile range, circles show individual termites, and black diamonds show means.

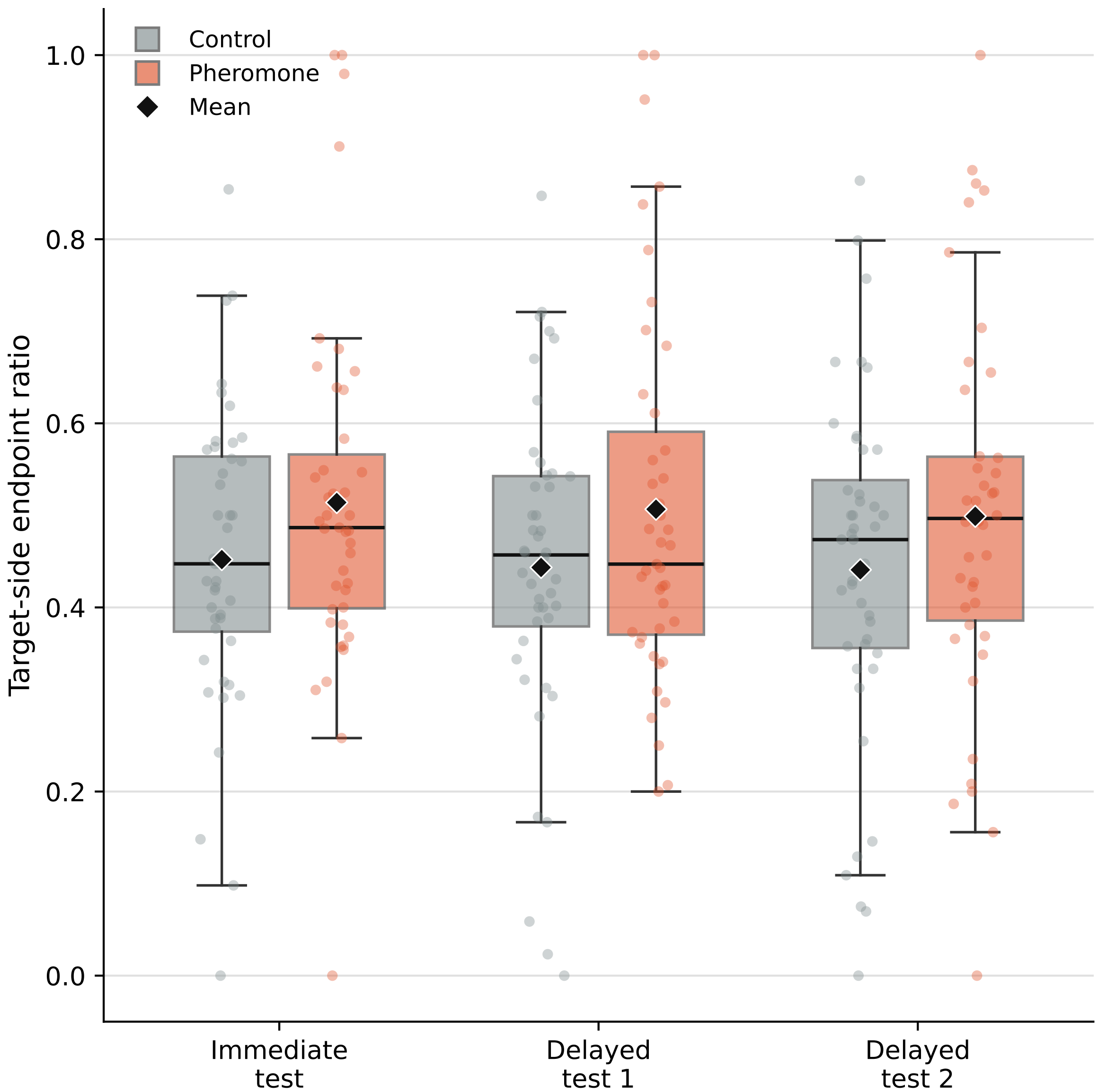

Fig B Target-side endpoint ratio for center-directed vectors. Boxes show the interquartile range, horizontal lines show medians, whiskers show 1.5 times the interquartile range, circles show individual termites, and black diamonds show means.

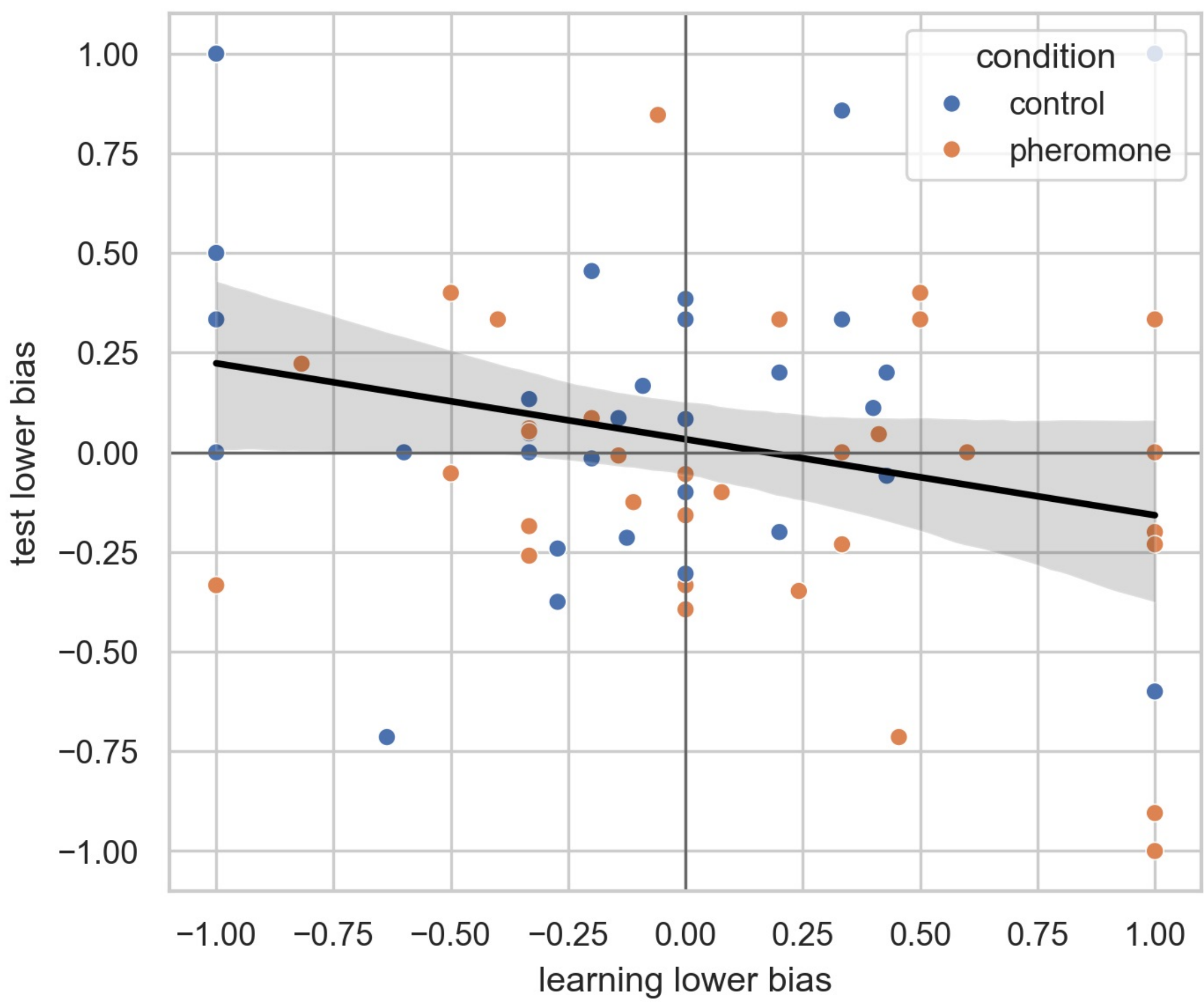

Fig C Relationship between learning-phase and test-phase target-side entry bias.

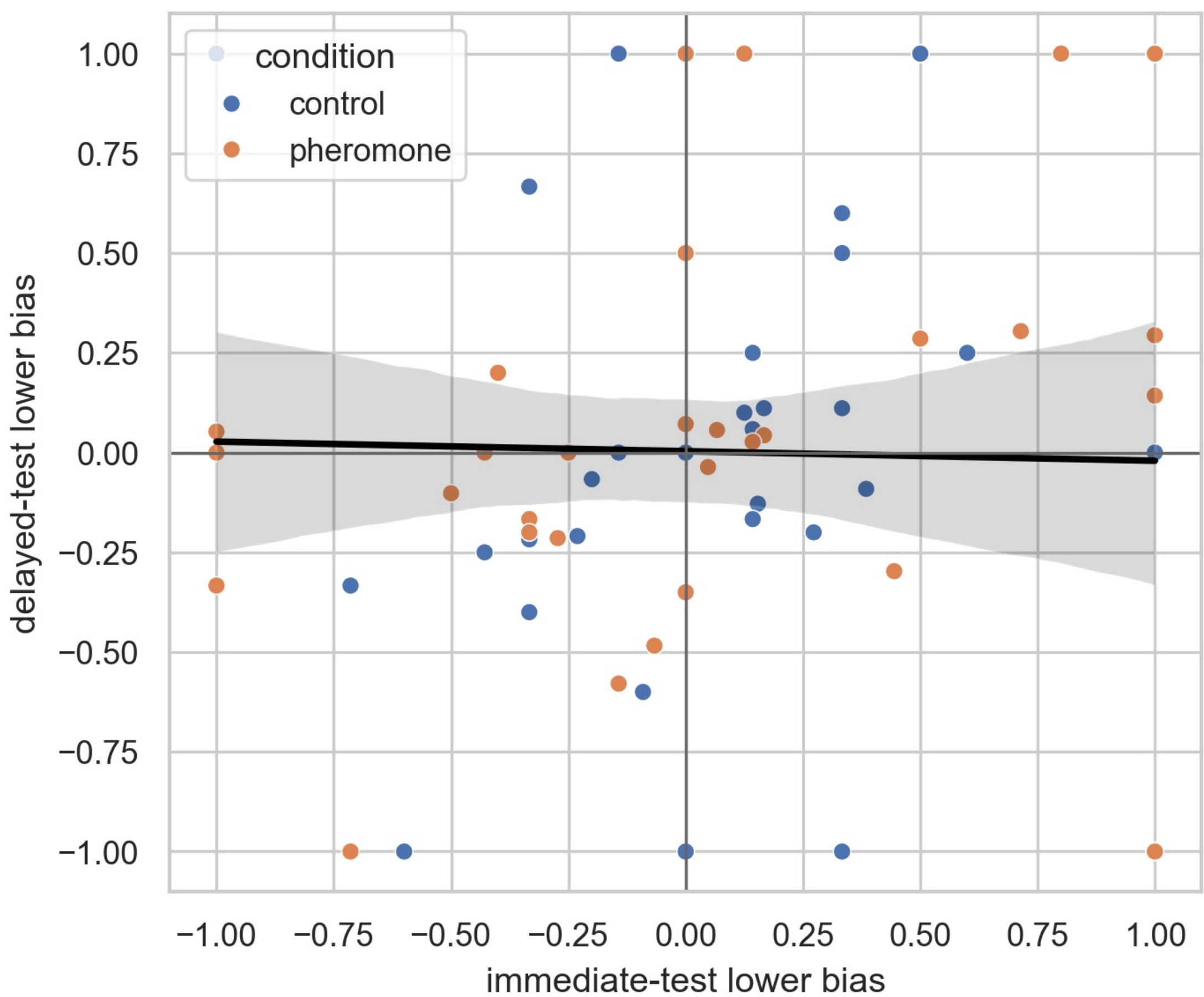

Fig D Relationship between immediate-test and delayed-test target-side entry bias.

Occupancy ratios

We also tested whether occupancy ratios differed between the target-side and opposite-side semicircles across phase and distance bins. After Benjamini-Hochberg false discovery rate correction, the clearest side effect was observed in the learning phase in the peripheral 35–42.5 mm bin ($q = 0.004$; Table E). Test-phase occupancy did not show a consistent target-side pattern after correction. This analysis therefore did not provide stronger evidence than the entry and excursion-event analyses.

Sensitivity analysis excluding termites without learning-phase target entry

As a sensitivity check, termites in the pheromone condition with zero entries into the target circle during the learning phase were excluded. In this subset, the test-phase target-entry model showed a positive pheromone effect (estimate = 1.836, SE = 0.904, $z = 2.032$, $p = 0.042$; Table F). The center-directed-vector count and endpoint-ratio analyses still did not provide strong evidence. This sensitivity analysis suggests that the target-entry result is not driven only by individuals that never approached the target during learning, but it is not used as the primary analysis because exclusion based on behavioral response can introduce selection bias.

Tables

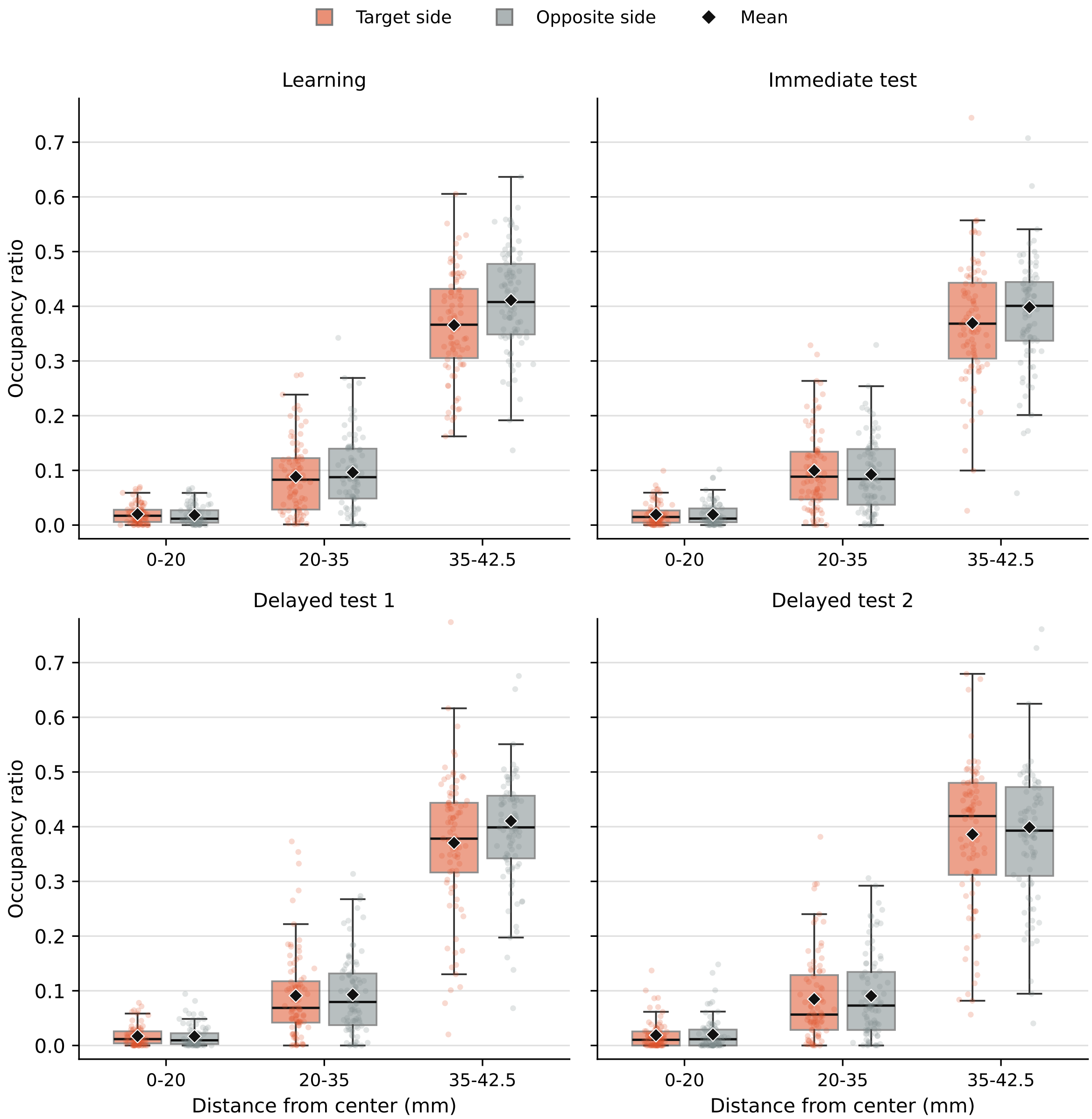

Fig E Occupancy ratios by side, phase, and distance bin. Boxes show the interquartile range, horizontal lines show medians, whiskers show 1.5 times the interquartile range, circles show individual termites, and black diamonds show means.

Table A. Fixed effects for target-side center-directed vector counts.

Term	Estimate	SE	z	p
Intercept	0.692	2.245	0.308	0.758
Pheromone condition	1.744	2.754	0.633	0.526
Delayed test 1	-0.882	0.754	-1.170	0.242
Delayed test 2	-2.089	0.755	-2.766	0.006
Total vectors	0.463	0.020	23.550	< 0.001

Table B. Fixed effects for target-side endpoint ratio.

Term	Estimate	SE	z	p
Intercept	0.459	0.033	13.982	< 0.001
Pheromone condition	0.066	0.035	1.886	0.059
Delayed test 1	-0.008	0.018	-0.425	0.671
Delayed test 2	-0.009	0.019	-0.489	0.625
Total vectors	-0.0001	0.0003	-0.413	0.680

Table C. Fixed effects for test-entry bias predicted by learning-entry bias.

Term	Estimate	SE	z	p
Intercept	0.000	0.159	0.000	1.000
Learning-entry bias	-0.173	0.087	-1.991	0.046
Total activity	0.000003	0.000005	0.669	0.503

Table D. Fixed effects for delayed-entry bias predicted by learning and immediate-test bias.

Term	Estimate	SE	z	p
Intercept	0.000	0.246	0.000	1.000
Learning-entry bias	-0.096	0.128	-0.749	0.454
Immediate-entry bias	0.078	0.129	0.606	0.544
Total activity	-0.000003	0.000007	-0.420	0.675

Table E. Side tests for occupancy ratios after FDR correction. Positive estimates indicate greater opposite-side occupancy than target-side occupancy.

Phase	Distance bin	Estimate	SE	z	p	q
Learning	0–20	-0.0017	0.0011	-1.538	0.124	0.372
Learning	20–35	0.0078	0.0060	1.306	0.192	0.383
Learning	35–42.5	0.0456	0.0127	3.582	< 0.001	0.004
Immediate	0–20	-0.0001	0.0010	-0.070	0.944	0.944
Immediate	20–35	-0.0072	0.0069	-1.043	0.297	0.509
Immediate	35–42.5	0.0291	0.0175	1.661	0.097	0.372
Delayed 1	0–20	-0.0003	0.0009	-0.334	0.739	0.886
Delayed 1	20–35	0.0019	0.0084	0.231	0.817	0.892
Delayed 1	35–42.5	0.0399	0.0191	2.086	0.037	0.222
Delayed 2	0–20	0.0013	0.0009	1.418	0.156	0.375
Delayed 2	20–35	0.0057	0.0084	0.674	0.500	0.735
Delayed 2	35–42.5	0.0127	0.0214	0.596	0.551	0.735

Table F. Sensitivity analysis excluding pheromone-condition termites with zero learning-phase target entries.

Analysis	Estimate	SE	z	p
Center-directed vector count	-0.013	3.112	-0.004	0.997
Endpoint ratio	0.034	0.037	0.908	0.364
Test-phase target-entry count	1.836	0.904	2.032	0.042
